# Using in-house 3D technology for optimal spatial positioning of elongation devices for distraction osteogenesis—a cost-effective alternative

**DOI:** 10.3389/froh.2025.1514050

**Published:** 2025-02-13

**Authors:** Adi Rachmiel, Dekel Shilo, Ahmad Hija, Tal Capucha, Nidal Zeineh, Omri Emodi, Andrei Krasovsky

**Affiliations:** ^1^ Ruth and Bruce Rappaport Faculty of Medicine, Technion-Israel Institute of Technology, Haifa, Israel; ^2^ Department of Oral and Maxillofacial Surgery, Rambam Health Care Campus, Haifa, Israel

**Keywords:** virtual surgical planning (VSP), distraction osteogenesis (DO), mandibular retrognathism, patient specific 3D implants, in house production

## Abstract

**Introduction:**

Mandibular distraction osteogenesis is globally accepted as the gold-standard surgical solution for various craniofacial deformities and syndromes. Stock device evolution has advanced into complex designs to achieve the most accurate three-dimensional distraction vector of elongation. Today's cutting-edge solution is patient-specific distractors designed by virtual surgical planning (VSP) to facilitate surgical performance and ensure the most predictable clinical results. However, tailoring patient-specific distractors comes with a significant price tag.

**Methods:**

Using VSP technology, we developed an inexpensive stepwise method of precisely directing the distraction vector by adapting off-the-shelf distractors for the individual contour of the patients’ mandibles based on the in-house designed and printed cutting guides.

**Results:**

The virtual planning sequence and clinical application are described in detail. The final results are assessed by 3D analysis to confirm the method's precision and predictability.

**Discussion:**

The final positions of the adapted off-the-shelf distractors were found to match the pre-operative virtual planning precisely, resulting in excellent clinical results. This method can be easily reproduced in similar clinical cases with reduced cost.

## Introduction

Mandibular distraction osteogenesis is widely used to treat various congenital craniofacial abnormalities and acquired traumatic conditions. Some of the most common are Pierre-Robin sequence (PRS), hemifacial microsomia (HFM), Treacher-Collins syndrome (TCS), and temporomandibular joint (TMJ) ankylosis (1, 2). Different distractors are available on the market, with the main distinction being external and internal devices. Internal distraction devices (IDD) are recommended as the first choice due to higher compliance, better stability, a more predictable vector of lengthening, and reduced scarring, despite the main disadvantage of a second surgical intervention for removal (3). The evolution of IDDs encompasses unidirectional, bidirectional, multidirectional, and curvilinear vectors of distraction, all in an attempt to reproduce the deficient segment in the most accurate form (4).

The control of the lengthening vector also directly depends on the precise intraoperative fixation of the IDD across the osteotomy plane of the mandible. To facilitate the intraoperative positioning of the IDD, patient-specific distractors, designed in a virtual surgical planning (VSP) environment, are now available on the market (5). The drilling holes of patient-specific cutting guides guide the positioning of patient-specific IDD. The exact direction and distance of the displaced distal segment can now be virtually planned, similarly to standard orthognathic surgery.

Despite the clear advantage of using patient-specific devices, the significantly higher prices for such technology may discourage low-budget medical facilities. A technical solution for much more affordable prices, providing similar clinical results, can expand the toolbox of many surgeons worldwide.

## Materials and methods

This is a retrospective study of patients with retrognathic mandible due to various etiologies treated in our department in 2024 using a novel VSP protocol. Inclusion criteria were symmetric bilateral mandibular retrognathism, planned mandibular advancement for 10 mm or more, patients with permanent dentition, and individuals without mandibular third molars. Exclusion criteria included patients with mixed and primary dentition, as well as patients with low postoperative compliance potential. The primary objective of this project was to develop a practical way to incorporate off-the-shelf internal curvilinear distractors into a VSP sequence, enabling patient-specific adaptation of the IDDs. Subsequently, an assessment of the method's accuracy was conducted.

**Figure 1:**
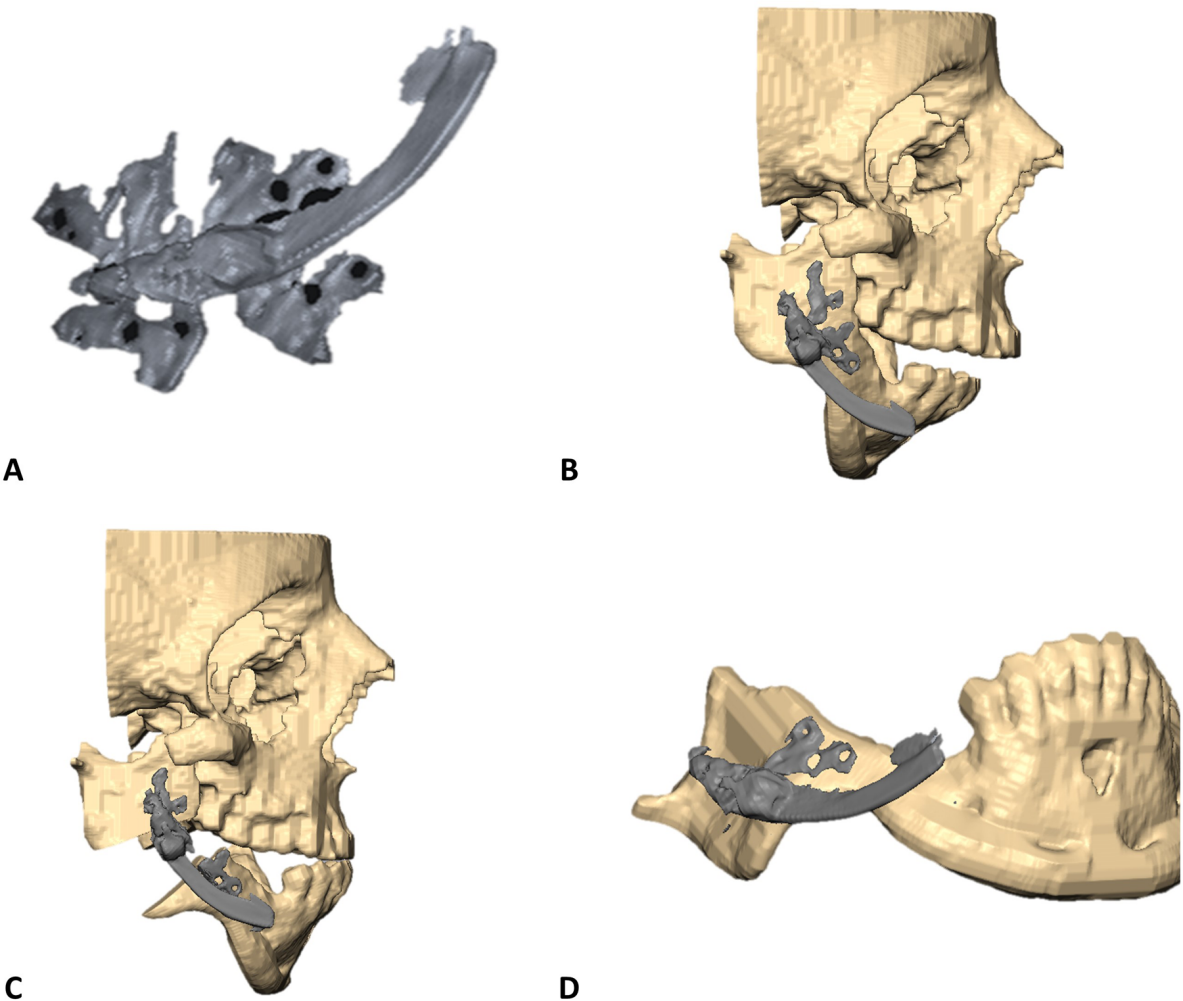
STL models orientation in VSP environment. **(A)** STL model of right-side internal curvilinear distractor device (DePuy Synthes) as segmented from CBCT DICOM. **(B)** Right-side IDD is positioned in the desired vector over the mandibular right angle. **(C)** Prediction of mandibular distal segment final position following the trajectory of elongation over the curved track of the IDD to a desired final occlusion. **(D)** Bent anchoring footplates continuous with the mandibular cortex.

**Figure 2:**
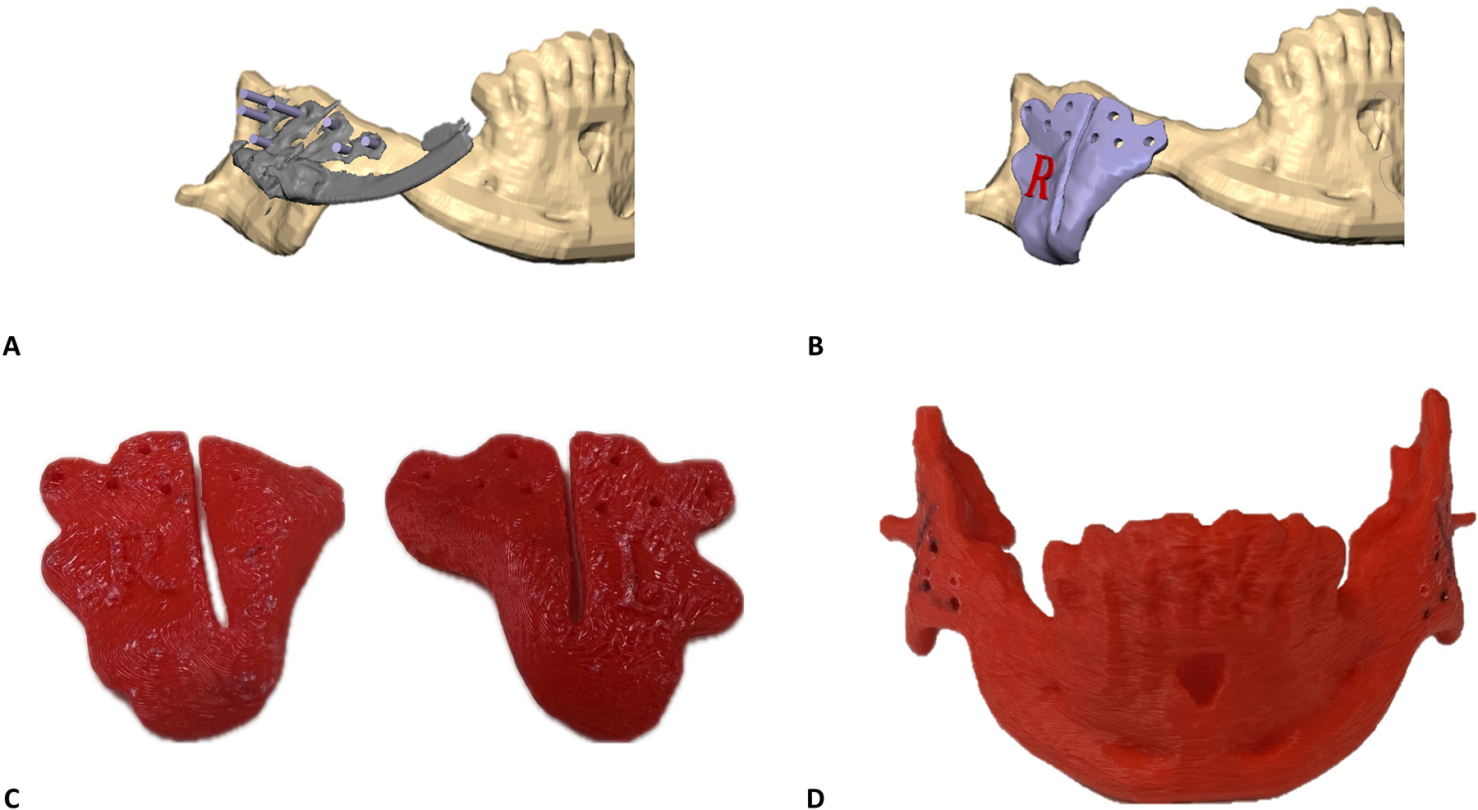
Surgical cutting guides customization principles: **(A)** surgical guides are designed to guide the osteotomy and mark the position of the drill holes for the ﬁnal position of the IDD. Purple rods represent the drilling holes’ angle and diameter to match the fixating holes of the bent anchoring footplate. **(B)** Surgical cutting guide for the final design of the right side. **(C)** In-house printed right and left surgical cutting guides. **(D)** In-house printed mandible containing the drilling holes to direct the bending of the anchoring footplates.

Cone Beam Computed Tomography (CBCT)-based Digital Imaging and Communications in Medicine (DICOM) files of the patients were imported into D2P (DICOM to Print) segmentation software (3D Systems, OR, USA) to create 3D STL files of the mandible and the maxilla in their preoperative anatomical relation. Next, the IDD was scanned using the same CBCT device. This study used an internal curvilinear distraction system (DePuy Synthes) for all patients. The DICOM file of the IDD was also imported into D2P for segmentation (Figure 1). STL files (facial bones and IDD) were imported into 3D planning Geomagic Freeform (3D Systems) software. The STL of the IDD was mirrored to create the contralateral virtual IDD. Both IDDs were virtually positioned along the mandibular angles in the optimal bilateral direction to produce the desired net vector of lengthening (Figure 1B). Particular effort was put into positioning the IDDs in the most parallel position to each other to secure maximum stability of the devices after activation and prevent deviation of the predetermined elongation vector resulting from contralateral unbalanced counterforces. The displacement of the distal segment was virtually simulated along the curved track of the distractor device to reach the desired occlusal relation (Figure 1C). Various curvature radii are available, allowing optimal elongation vectors and distance choice. Since the anchoring footplates of the stock device are not naturally aligned with the cortical surface of the mandible, the next essential step in the protocol was to virtually bend the relevant part of the anchoring footplates, which was performed in Geomagic Freeform until optimal surface alignment was achieved. The excess non-functional parts of the anchoring footplates were removed (Figure 1D). The design of the osteotomy line should cross between the opposing anchoring footplates. Directed by the chosen osteotomy line, a surgical cutting guide was designed so that the drilling holes of the guide matched the fixation holes of the virtually bent anchoring footplates of the IDD (Figures 2A,B). The mandible model (with the drilling holes) and the cutting guides were printed for intraoperative use (Figures 2C,D). Preoperatively, the right and left IDDs were positioned over the drilling holes on both sides of the mandible model. Anchoring footplates were manually bent using bending pliers until fixating holes optimally matched the drilling holes on the mandibular model. The remaining non-functional parts of the anchoring footplates were finally trimmed using a cutter. A 3D analysis was performed to compare the planned position and postoperative position of the IDDs using CloudCompare (Open source, Freeware). This method for 3D evaluation was described in our previous article (6).

## Results

In this study, three patients with severe mandibular retrognathism underwent distraction osteogenesis surgery. The etiologies for mandibular retrognathism were TCS (*n* = 1), bilateral HFM (*n* = 1), and idiopathic condylar resorption (ICR) (*n* = 1). Patients’ mean age was 16.7 years. The preoperative VSPs were performed for all cases according to a described protocol, and postoperative 3D accuracy evaluations were conducted.

**Figure 3:**
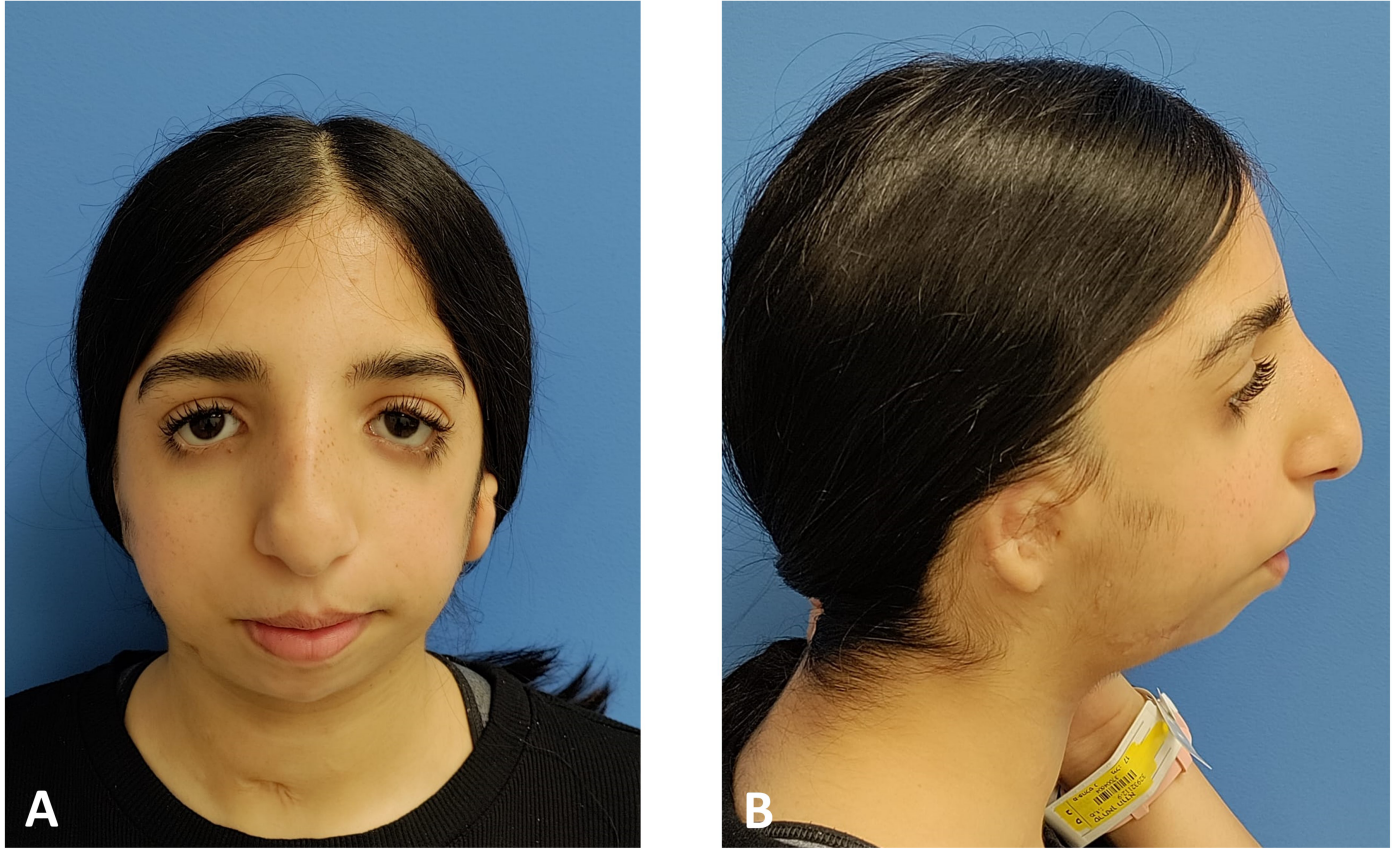
Preoperative clinical extraoral photographs: **(A)** en face typical view of a patient with TCS. **(B)** The mandible is severely retruded, as seen in the profile view.

**Figure 4:**
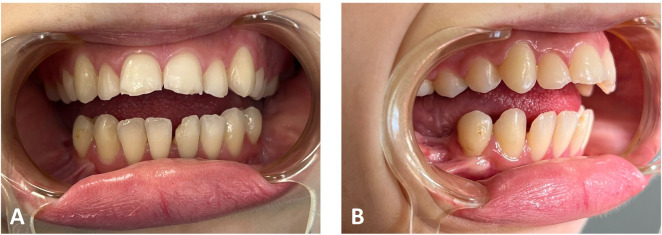
Preoperative clinical intraoral photographs: **A** and **B**. Open bite and increased overjet.

**Figure 5:**
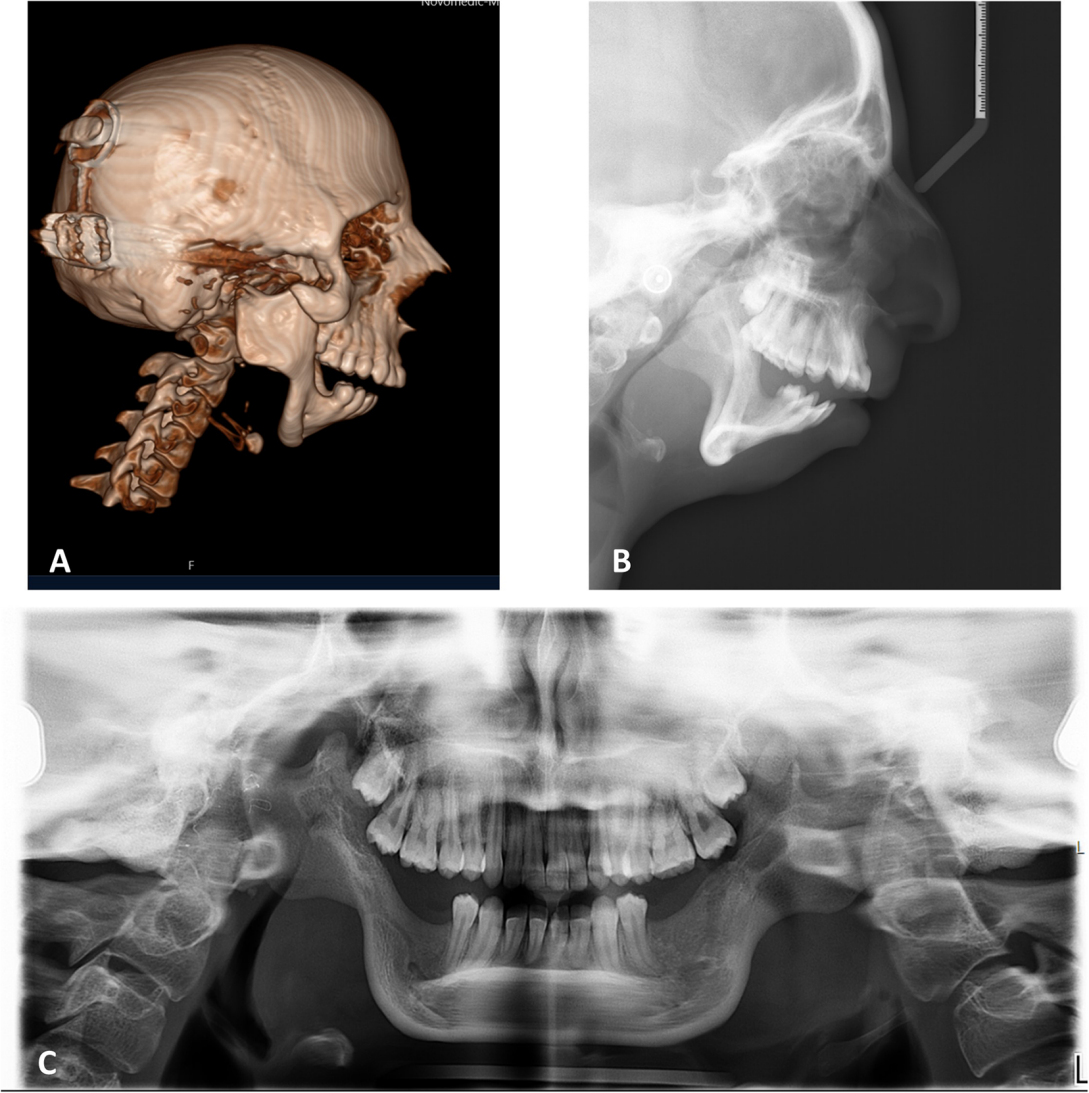
Preoperative radiography. **(A)** Sagittal view of CBCT and lateral cephalometry **(B)** demonstrate the retruded position of the pogonion, short ramie, missing condyles, deep mandibular antegonial notches, all associated with TCS phenotype. **(C)** Panoramic radiograph emphasizing the symmetry of the condition.

**Figure 6:**
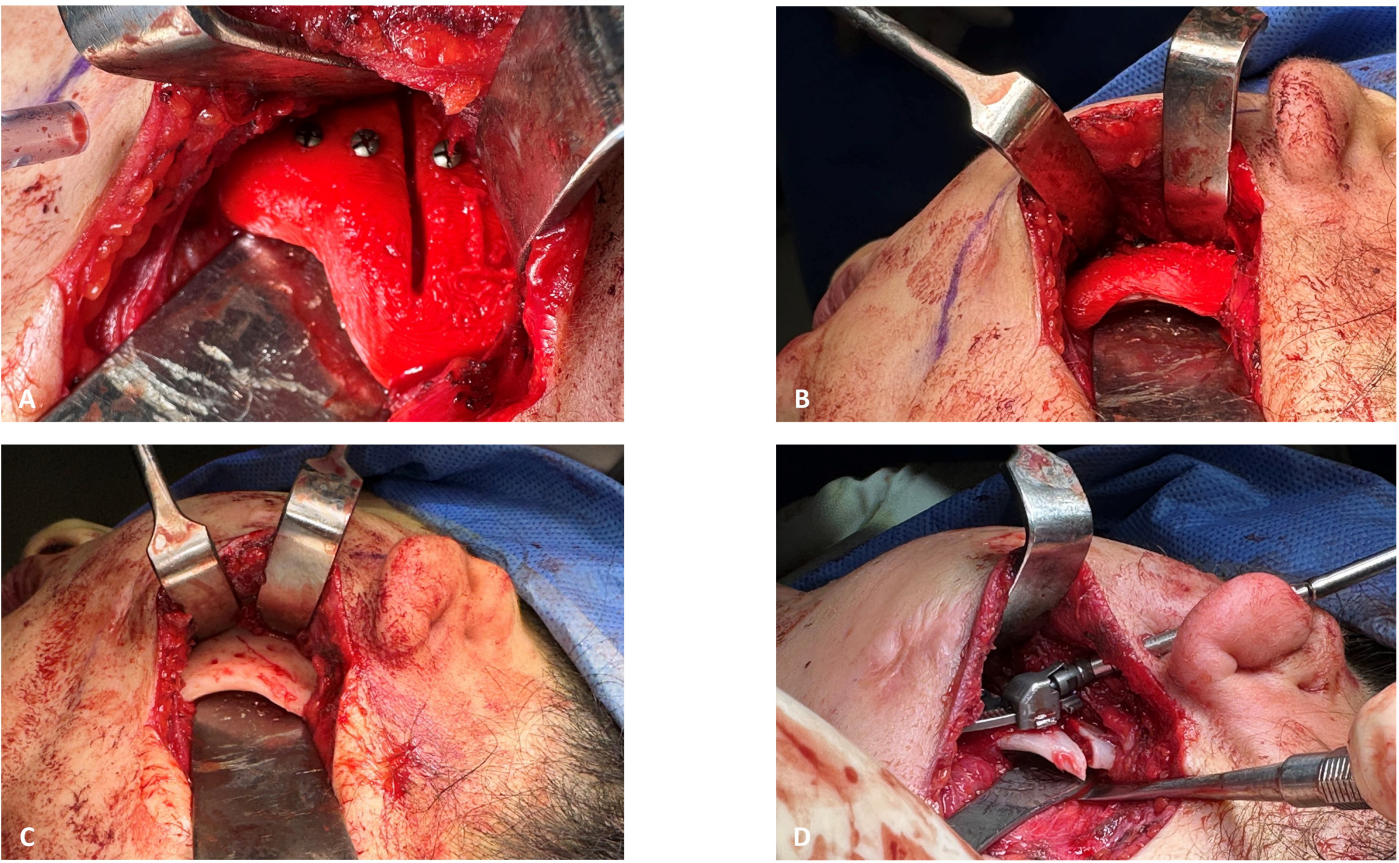
Intraoperative view: **(A)** left surgical gutting guide fixated with screws to guide the osteotomy line. **(B)** Inferior view of the left surgical guide fit to the mandibular angle. **(C)** Fixation holes for left IDD and the mark of the osteotomy line, made through the surgical cutting guide, as seen after it was removed. **(D)** Fixated left IDD after completion of the osteotomy.

Clinical and analytical results of a patient with TCS are presented next for explanatory purposes in practical protocol implementation. Clinical photographs and radiographic data were collected in the first step. Figures 3, 4 illustrate the open bite malocclusion caused by a deficient mandible before surgical intervention. CBCT, lateral cephalometric, and panoramic radiographs demonstrate a short, thin ramie and the ultimate absence of the condyles (Figure 5). Intraoperatively, the Submandibular (Risdon) approach was used to expose the mandibular angles and ramie. In-house surgical cutting guides were fixed in place using screws after being confirmed to be correctly positioned without any rocking movement (Figures 6A,B). The cutting guides were removed after fixating holes were drilled, and the osteotomy lines were marked (Figure 6C). Following the fixation of the IDDs, the osteotomies were performed using the key steps first described by Rachmiel et al. (7) (Figure 6D).

**Figure 7:**
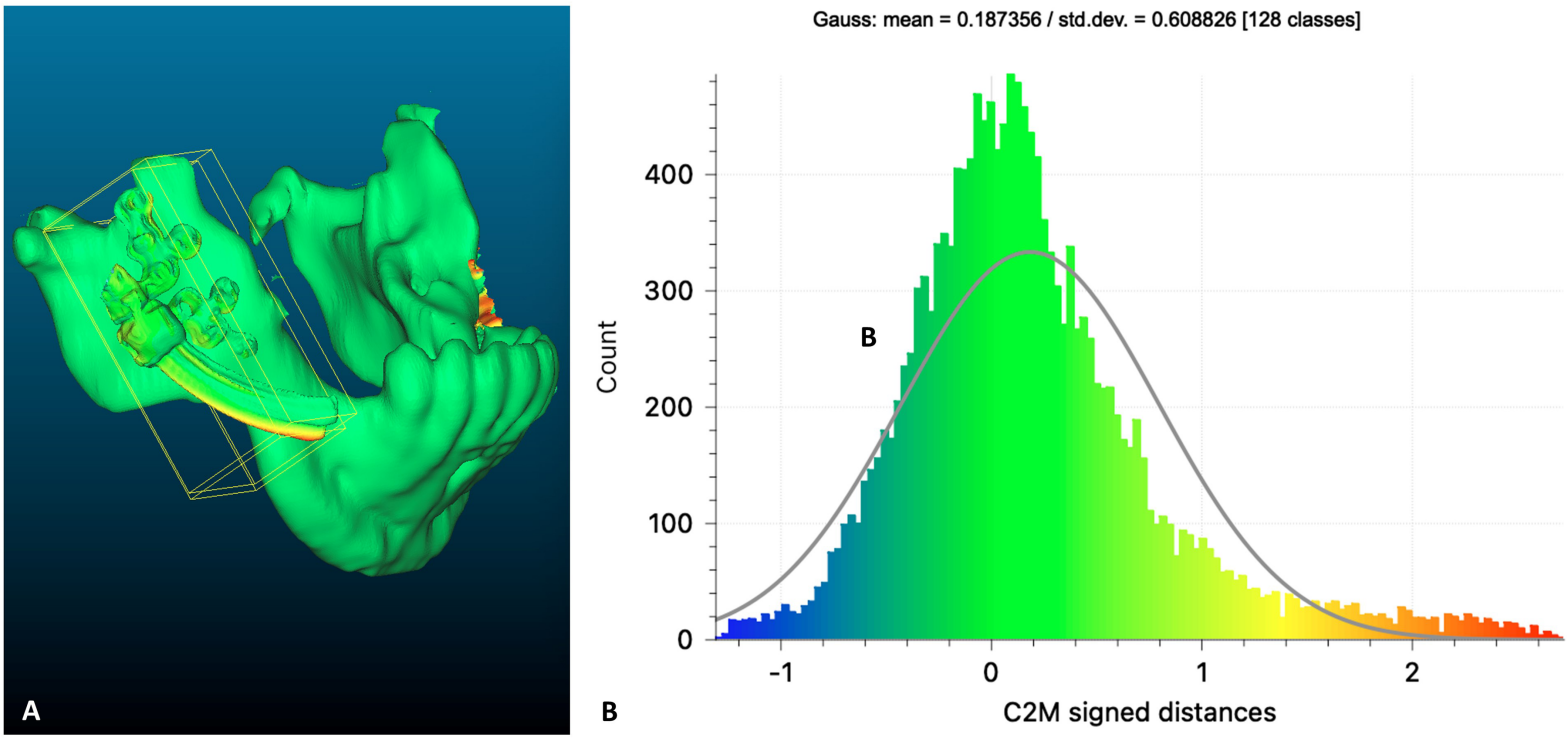
Planned vs. postoperative: **(A)** heat map demonstrating differences between a preoperative (green) virtual planning of the right IDD positioning over the mandible compared to postoperative result (yellow). **(B)** Distribution of the distances between the planned and the postoperative position of the right IDD.

The patient completed a postoperative CBCT on the next day. The derived postoperative STL model (mandible and IDD) was superimposed on the previous virtually planned model to evaluate the accuracy of the spatial fixation of the IDD on the mandible. In Figure 7A, the superimposed result of the mandible and the right-side IDD is shown. The green color represents the preoperatively planned position of the mandible and the IDD. The postoperative STL is represented by a yellow color, where only the curved track of the distractor can be noticed, indicating the extent of deviation from the virtual plan. The results of the 3D analysis showed an average distance deviation of 0.187 mm between the virtually planned and postoperative IDDs (Figure 7B). Similarly, the mean distance on the left side was 0.127 mm (not shown).

**Figure 8:**
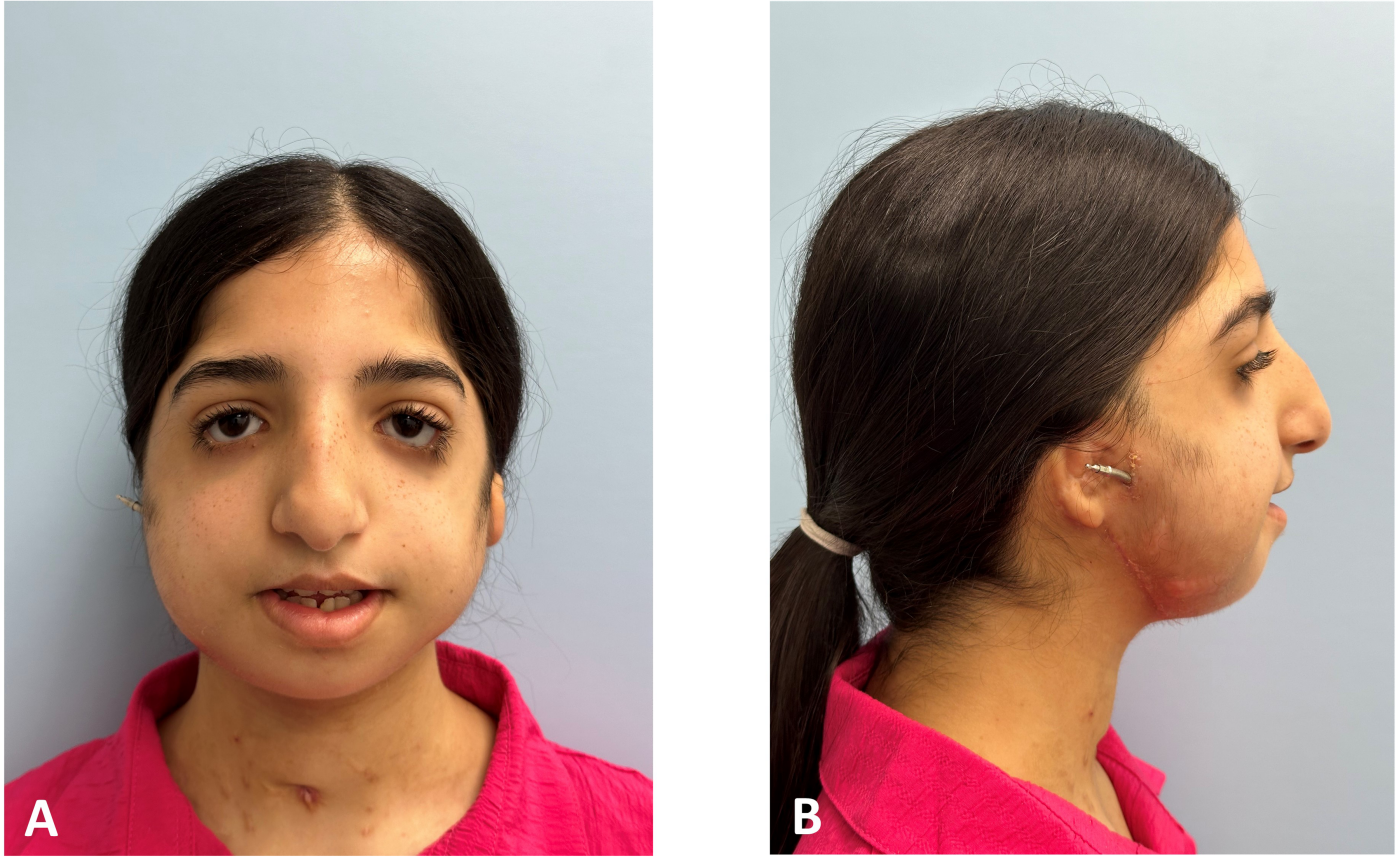
Post-activation clinical extraoral photographs: **(A,B)** improved anterior position of the mandible with an increased vertical height of the lower third.

**Figure 9:**
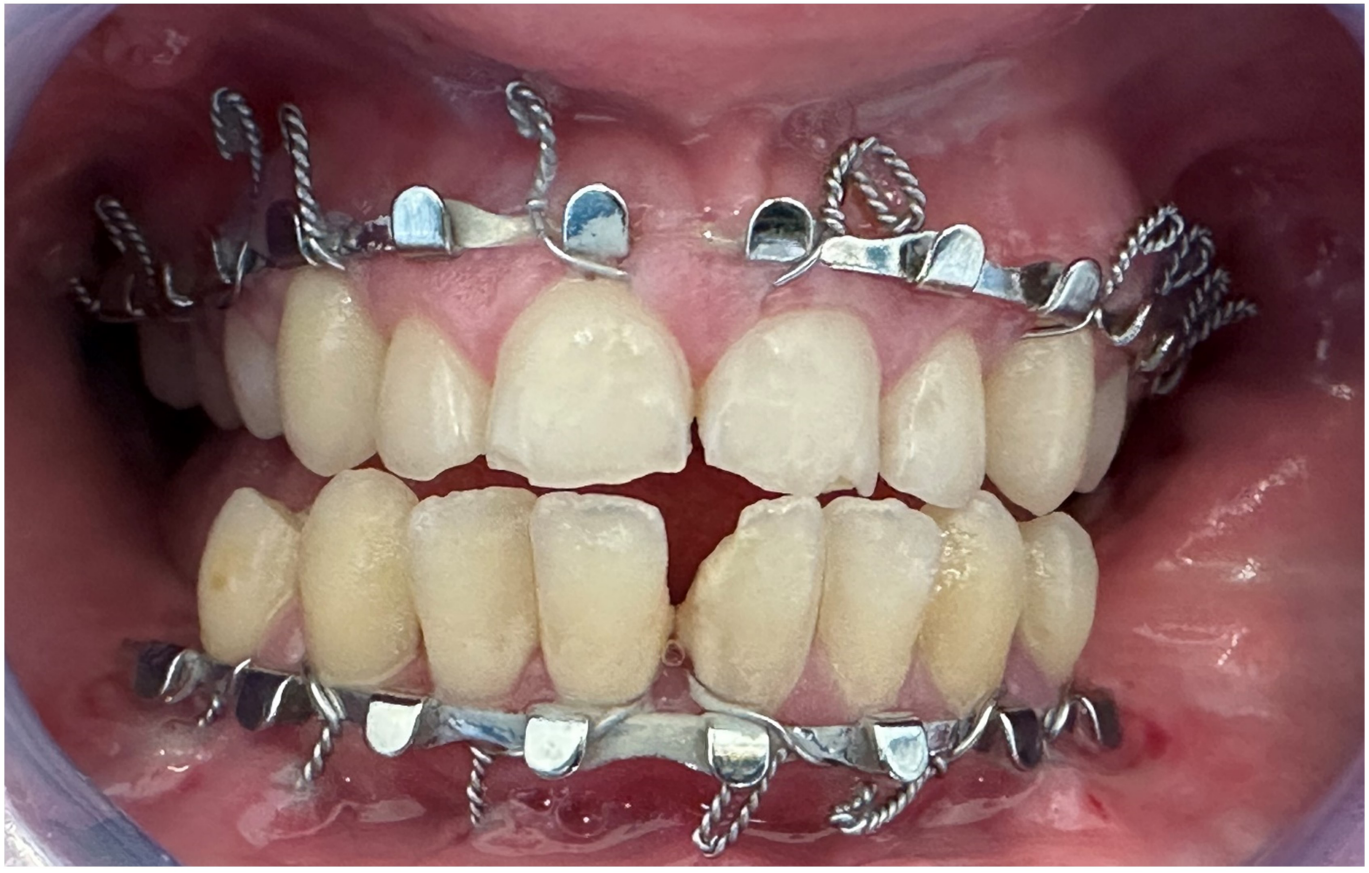
Post-activation clinical intraoral photographs: closed open bite and overcorrected overjet. The upper and lower midlines are continuous.

**Figure 10:**
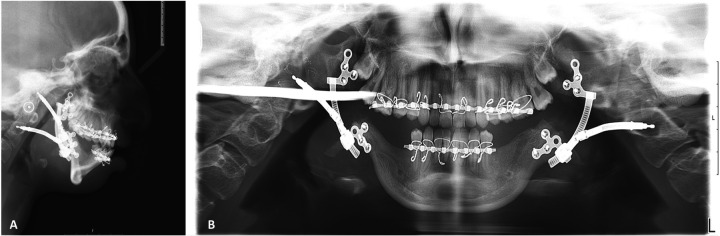
Post-activation radiography: **(A)** lateral cephalometry demonstrating overcorrected negative overjet, in close similarity to virtual planning (Figure 1C), improved position of pogonion, and increased airway. **(B)** Panoramic radiograph demonstrating the symmetry of bilateral elongation.

Clinical and radiographic results at the end of the activation phase shown in Figures 8–10 demonstrate changes in the esthetic profile and occlusal relation.

## Discussion

The existing literature already documents the utilization of computer-aided design (CAD) and computer-aided manufacturing (CAM) technology as a supplementary tool in distraction osteogenesis surgeries of the mandible using stock devices (8). However, to our knowledge, this is the first time an off-the-shelf IDD has been imported into a VSP system to direct and predict the desired displacement of the distal segment of the mandible. Furthermore, the process of customizing patient-specific cutting guides for accurate positioning and fixation of the stock IDDs has not been documented. Literature finds distraction osteogenesis superior for mandibular advancement of 10 mm or more compared to sagittal split osteotomy, as it achieves greater long-term stability and reduces relapse rates (9). Correct parallel fixation of the IDDs using VSP will further reduce relapse by achieving a symmetrical elongation, resulting in more stable occlusion. Tooth injury and inappropriate distraction vector setting are some of the complications anticipated in mandibular distraction osteogenesis, which can be as high as 22.5 and 8.8 percent, respectively (10). Using patient-specific cutting guides helps avoid vital structure injuries, especially teeth buds, in growing patients. Specific complications related to the VSP sequence during mandibular distraction osteogenesis should be recognized and prevented. Equivocal positioning of rocking cutting guides will lead to improper osteotomy lines and misaligned fixating screw holes, resulting in a nonparallel position of the IDDs. The broader contact surface of the cutting guide with the mandibular cortex will ensure a single stable positioning but on the expanse of more extensive periosteal stripping. Precise manual bending of the footplates over the mandibular printed model can become challenging, but maximal contact must be achieved. Fixating IDD with floating footplates will lead to the displacement of the device during screw insertion. We found no difference in complications related to the patients’ etiologies. In our experience, complications can arise mainly due to the inaccuracy of the surgical technique and patient compliance during the postoperative period. Implementing the VSP sequence improves surgical technique. However, difficulties with patient compliance may still occur during the activation period when bone segments are gradually pulled apart, which may be accompanied by some degree of pain. From our experience, we find compliance related to patients’ personalities and family support. The postoperative course also includes elastics and posterior build-ups for directing and stabilizing the occlusion, for which a certain level of patient cooperation is again required. Finally, Le-Fort 1 osteotomy with maxillary advancement and counterclockwise rotation is usually necessary to achieve a natural facial profile and stable class 1 occlusion.

The results of this study qualitatively and quantitatively demonstrate the precision of translating the virtual planning position into physical intraoperative fixation of IDDs (Figure 7), thus reducing the incidence of improper vectors, which is considered the most troublesome complication. Optimally, the distractors must be placed as parallel as possible. Otherwise, possible subsequent bending of the distractor parts will harden the turning motion and eventually may jam to stop the elongation mechanics. Our protocol allows for bilateral virtual paralleling of the IDDs to overcome this challenge. The symmetry of bilateral elongation was perfectly maintained in the treated patients, as demonstrated by the continuation of the upper and lower dental midlines (Figures 9, 10B). Finally, the described protocol predictability was also confirmed clinically, as can be seen by comparing the prediction of the final position of the distal segment of the mandible (Figure 1C) with the lateral cephalometric radiograph at the end of the activation phase (Figure 10A). Virtual surgical planning for distraction osteogenesis can be similarly utilized in patients with significant maxillary deficiency and cases of unilateral asymmetry. This treatment approach is applicable in Oral and Maxillofacial Departments equipped with basic 3D planning and printing technology and relevant surgical experience in distraction osteogenesis of the maxillofacial complex.

Using the presented VSP protocol significantly reduces the overall cost of the procedure. Outsourcing surgical planning, printing cutting guides, and manufacturing patient specific IDDs can cost up to $27,500 for a single bilateral case. In contrast, a high-quality Fused Deposition Modeling (FDM) printer can be purchased for around $3,000, enabling long-term in-house production of cutting guides. The consumables needed for printing the mandible and cutting guides are minimal, costing only a few dollars per case. Additionally, a bilateral stock internal curvilinear distraction system from DePuy Synthes for the mandible is priced at approximately $10,000. As a result, a total cost reduction of more than 50% is anticipated.

To conclude, this work proposes a novel, low-cost, predictable VSP protocol to achieve better clinical outcomes in mandibular distraction osteogenesis when using off-the-shelf distractors. Although patient-specific distractors are setting the highest benchmark in the field, other, more affordable options with comparable results are valuable additions to the armamentarium of craniofacial surgery.

## Data Availability

The original contributions presented in the study are included in the article/Supplementary Material, further inquiries can be directed to the corresponding author.
